# Humidity-dependent surface tension measurements of individual inorganic and organic submicrometre liquid particles†
†Electronic supplementary information (ESI) available: SEM image of AFM nanoneedles, force plot data from bulk AFM surface tension, data used for surface tension *vs.* RH predictions, comparison of AIM and bulk predictions for NaCl. See DOI: 10.1039/c4sc03716b



**DOI:** 10.1039/c4sc03716b

**Published:** 2015-03-31

**Authors:** Holly S. Morris, Vicki H. Grassian, Alexei V. Tivanski

**Affiliations:** a Department of Chemistry , University of Iowa , Iowa City , Iowa 52242 , USA . Email: vicki-grassian@uiowa.edu ; Email: alexei-tivanski@uiowa.edu

## Abstract

Surface tension, an important property of liquids, is easily measured for bulk samples. However, for droplets smaller than one micron in size, there are currently no reported measurements. In this study, atomic force microscopy (AFM) and force spectroscopy have been utilized to measure surface tension of individual submicron sized droplets at ambient pressure and controlled relative humidity (RH). Since the surface tension of atmospheric aerosols is a key factor in understanding aerosol climate effects, three atmospherically relevant systems (NaCl, malonic and glutaric acids) were studied. Single particle AFM measurements were successfully implemented in measuring the surface tension of deliquesced particles on the order of 200 to 500 nm in diameter. Deliquesced particles continuously uptake water at high RH, which changes the concentration and surface tension of the droplets. Therefore, surface tension as a function of RH was measured. AFM based surface tension measurements are close to predicted values based on bulk measurements and activities of these three chemical systems. Non-ideal behaviour in concentrated organic acid droplets is thought to be important and the reason for differences observed between bulk solution predictions and AFM data. Consequently, these measurements are crucial in order to improve atmospheric climate models as direct measurements hitherto have been previously inaccessible due to instrument limitations.

## Abstract 2

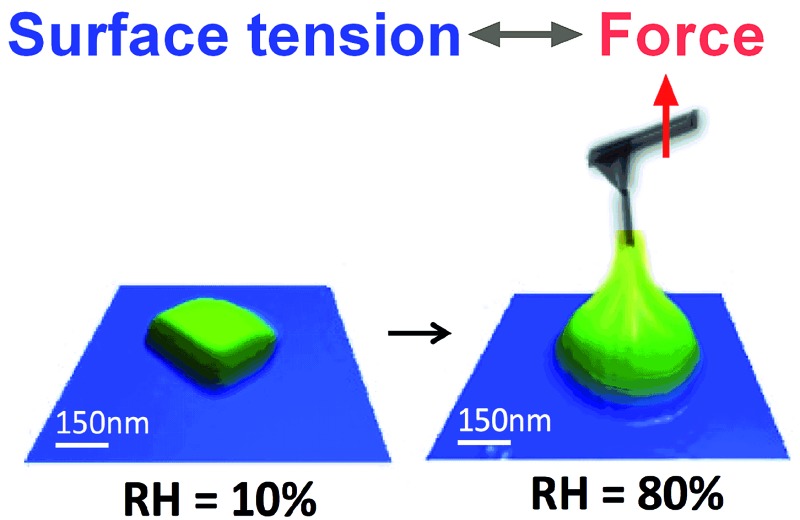
Atomic force microscopy has been utilized to measure the surface tension of atmospherically relevant droplets smaller than one micron.

## Introduction

The effect of aerosols on the earth's climate represents one of the biggest areas of uncertainty and understanding of factors that control our environment. Particles and liquid droplets in the atmosphere are chemically diverse,^1^ and they influence the radiative balance by scattering and absorbing solar radiation, and play an important role in cloud formation.^2,3^ One important property of aerosol droplets is surface tension, which is a key component in Köhler theory and climate models.^2,4–6^ For example, eqn (1) is the Kappa-Köhler expression that is utilized to determine the supersaturation ratio, *S*, over a droplet.^7^ The value of surface tension of the droplet (*σ*) is an important component of the exponential term (other parameters are: *d* = droplet diameter, *D*_p_ = dry particle diameter, *κ* = hygroscopicity parameter, *M*_w_ = molecular weight of water, *R* = gas constant, *T* = temperature, *ρ*_w_ = density of water).1
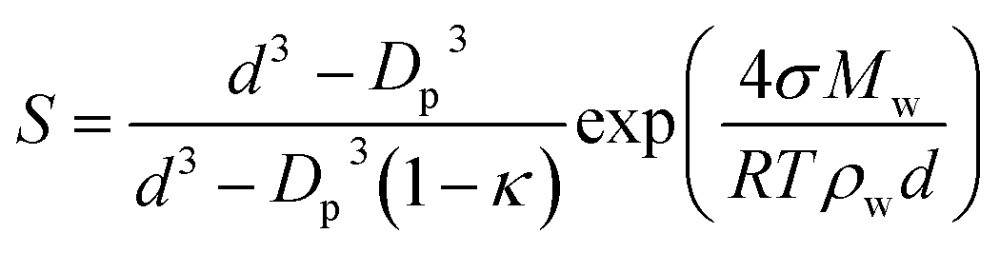



Surface tension depression of aqueous particles relative to pure water due to the presence of organic species can alter the aerosol's ability to act as cloud condensation nuclei (CCN).^4,8,9^ Phenomena that may occur in submicron size liquid droplets such as interior concentration depletion due to surface partitioning of organic species^4,9^ and size effects^10^ are still not well understood. Importantly, as addressed in detail here, there are no experimental methods that can directly measure the surface tension of droplets that are on the size range at or below a micrometre. Aerosols in this size range are highly important due to their prolonged lifetime in atmosphere.^11^ Consequently, surface tension values used in models are often times assumed to be that of pure water or that of concentrated bulk solution.^12^ Realistically, these assumptions can cause inaccuracies,^4^ especially when considering water-soluble chemical concentration, and therefore, surface tension change as a function of relative humidity (RH)^13,14^ in the atmosphere, which varies greatly depending on location and conditions.^15^

Particle size is a major factor and sometimes obstacle of understanding aerosol climate affects. Analysis of breaking ocean waves,^16^ as well as laboratory controlled wave action^17,18^ has shown that the majority of sea spray aerosol (SSA) particles are less than 1 μm in diameter.^19^ Considering this size scale, nanotechnology and nanoscopic methods are necessary in the effort to understand properties of SSA. Atomic force microscopy (AFM) has proven to be an excellent method for studying systems at the nanoscale.^20^ Highly resolved force sensing capabilities and sub-nanometre 3D spatial resolution make AFM a powerful investigation tool for studying nanoparticles. Vacuum conditions, which may alter particles, are not required, and experiments can be performed under controlled RH.^21^ While AFM is frequently used to study nanoparticle morphology^22^ and size,^23^ the efforts of this study were to develop AFM based methodology to measure the surface tension of submicron sized liquid droplets at controlled RH. Optical tweezers has been used to indirectly estimate the surface tension of micrometre sized NaCl droplets but the value observed is lower than expected, assumedly due to adsorption of trace species from the gas phase.^24^ AFM based surface tension measurements have been previously performed on bulk solutions,^25^ thin liquid layers,^26^ and micrometre sized oil droplets and bubbles in water.^27,28^ To the best of our knowledge, there are no studies that directly measure the surface tension of droplets smaller than several microns in size and furthermore, no studies that investigate surface tension as a function of RH of small, atmospherically relevant droplets.

For simplicity in developing this methodology, we have chosen single component chemical model systems of atmospheric relevance. NaCl is a major constituent of SSA and was chosen as an inorganic model system.^29,30^ Besides inorganic salts, organic enrichment in SSA is an important aspect of aerosol chemistry^31,32^ and greatly influences surface tension, thus atmospherically relevant organic compounds were selected as well. Low molecular weight dicarboxylic acids such as glutaric acid (GA) and malonic acid (MA) are prevalent chemical species in atmospheric particles.^33,34^ Dicarboxylic acids are water-soluble, surface active molecules and have been shown to alter hygroscopic properties of aerosols, which causes surface tension depression and changes in actual and predicted CCN activity.^35–38^ Thus, organic model systems used in the method development are GA and MA.

## Experimental

### Sample preparation

Aerosols were generated with a constant output atomizer (TSI, Inc., model 3076) from 100–200 mM aqueous stock solutions. All chemicals used were reagent grade (99.99% purity, Aldrich) and dissolved in deionized water (18 MΩ cm). The aerosol was passed through a diffusion dryer (TSI, Inc., model 3062), then size selected and deposited by impaction with a micro-orifice uniform deposit impactor (MOUDI) (MSP, Inc., model 110) onto hydrophobically coated silicon wafers.^39^ The particles in this study were collected on stage 6 of the MOUDI, which has an aerodynamic size cutoff of 1 μm at 50% collection efficiency and particle size range of 0.56–1.0 μm. In most cases, the substrate deposited particles were prepared and studied at the same day to avoid possible sample aging.^40^

### AFM based force spectroscopy

A molecular force probe 3D AFM (Asylum Research, Santa Barbara, CA) was used for all force spectroscopy and imaging. The silicon wafer containing particles was placed in a custom-made humidity cell,^21^ attached to the AFM head. High aspect ratio, constant diameter Ag_2_Ga nanoneedles (NN-HAR-FM60, NaugaNeedles) with nominal spring constant of 3.0 N m^–1^ were used for surface tension measurements and particle imaging. The AFM probe was first calibrated by determining the inverse optical linear sensitivity and spring constant with a thermal noise calibration method.^41^ The sample was imaged in AC mode to locate individual particles, then the RH was slowly raised, and force-distance plots were collected at the center of the droplet. After each change in RH, and before taking AFM measurements, the cell was allowed to equilibrate for *ca.* 15 minutes. For GA, the relative humidity was raised quickly at the beginning of the experiment, and then slowly decreased while performing the force spectroscopy at different RH on the dehydration cycle. A tip velocity of 1 μm s^–1^ was found to give the most stable force data and was used for all measurements. Approximately 20–30 force plots were collected at each RH on several individual droplets. The probe was either cleaned in deionized water between experiments or a new probe was used because crystals were observed (*via* electron microscopy) to solidify on the end of the needle after being subjected to the concentrated solutions, which effectively changes the diameter of the probe and therefore, the surface tension quantification.

### Bulk surface tension measurements

Bulk surface tension measurements were performed with a Kibron AquaPi tensiometer. The tensiometer was calibrated with deionized water before each use and the dyne probe was clean with ethanol, water and flame between experiments. All chemicals (NaCl, GA and MA) used were dissolved in deionized water (18 MΩ cm). Serial dilutions were performed to obtain surface tension as a function of a solute concentration.

## Results and discussion

### AFM surface tension measurements

Utilizing the AFM as a tensiometer was first reported by McGuiggan *et al.* in 2006, using a quartz rod.^42^ The production and commercialization of constant diameter Ag_2_Ga nanoneedles grown on AFM tips have made it possible to probe droplets as small as several hundred nanometres in size.^43^ The surface tension (*σ*) of individual submicron sized droplets is calculated by quantifying the retention force (*F*_Ret_) between the nanoneedle and the liquid droplet, and by knowing the radius (*r*) of the probe (eqn (2)), assuming that the liquid meniscus is parallel to cylindrical probe at maximum force.^25^

2 *F*_Ret_ = 2π*σr*

**Fig. 1:**
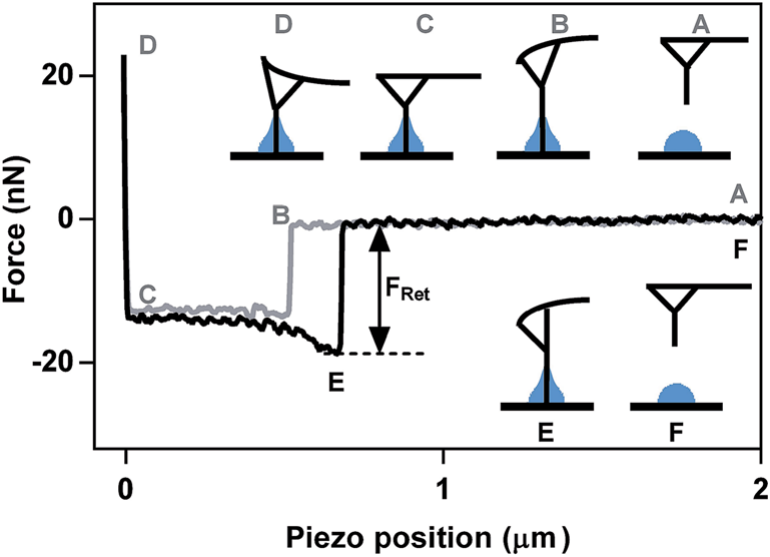
Typical AFM force plot measurement depicts the series of events that occur during approach (grey) and retract (black) cycle of the AFM cantilever on the submicron-sized droplet. The retention force (*F*_Ret_) is used to quantify the surface tension.

The retention force is the amount of force required to break the meniscus pinned at the end of the cylindrical probe from the liquid interface. A pictorial representation of an example force plot on a droplet is shown in Fig. 1. The needle starts at a position above the droplet (A) and then approaches the droplet vertically, in the *z*-direction, until it comes in contact with the liquid, causing spontaneous formation of a meniscus to rise on the cylinder, bending the probe downward, and resulting in a negative force (B). The horizontal portion of the force plot between B and C is a result of the needle moving through the liquid droplet and coming into contact with the substrate at point C. When a predefined maximum amount of force is reached, the tip retracts back away from the sample (D). At point E, the probe experiences a large attractive force due to the liquid meniscus holding the needle at the surface of the droplet. Once the meniscus is broken, the tip quickly retracts back to zero force and returns to an equilibrium distance above the sample. The retention force is defined as the absolute value of the difference in force when the tip jumps away from the droplet and back to zero force. The jump-away point occurs when the gradient of interaction forces becomes less or equal to the spring constant of the cantilever.

At the start of the experiment, a silicon wafer containing substrate-deposited particles is placed in a humidity cell and the RH is slowly raised. The phase transition from a solid particle to a liquid droplet (deliquescence) and the reverse transition (efflorescence) depend on chemical composition. The deliquescence point of NaCl and GA is a relatively sharp transition at approximately 75% (ref. 36) and 83–85% (ref. 11) RH, respectively. MA is highly hygroscopic and steadily uptakes water from as low as 10% RH.^11^ These hygroscopic properties dictated the range of RH that was probed for the surface tension experiments and are discussed in more detail below.

An initial important control study was performed to verify that bulk AFM surface tension measurements of pure water agree with bulk tensiometer results. For the AFM, a Petri dish containing ∼50 ml of DI water was placed on the AFM stage. Once the nanoneedle was in the vicinity of the water, force plots were collected, displaying profiles consistent with the needle contacting the liquid surface, resulting in large retention force (see ESI† for details). Based on 20–30 force measurements of the retention force and utilizing eqn (2), AFM based surface tension of water was determined to be 72.6 ± 0.5 mN m^–1^, which agrees within standard deviation with the bulk tensiometer value of 73.2 ± 0.1 mN m^–1^. Slight discrepancy between the two numbers is likely due to error associated with the value of the radius of the nanoneedle used in the AFM calculation, as this value is estimated from an SEM image. We note that such an experiment can be used as a calibration step to determine the exact diameter of the needle or to investigate if the tip is damaged or contaminated.

### NaCl AFM results

**Fig. 2:**
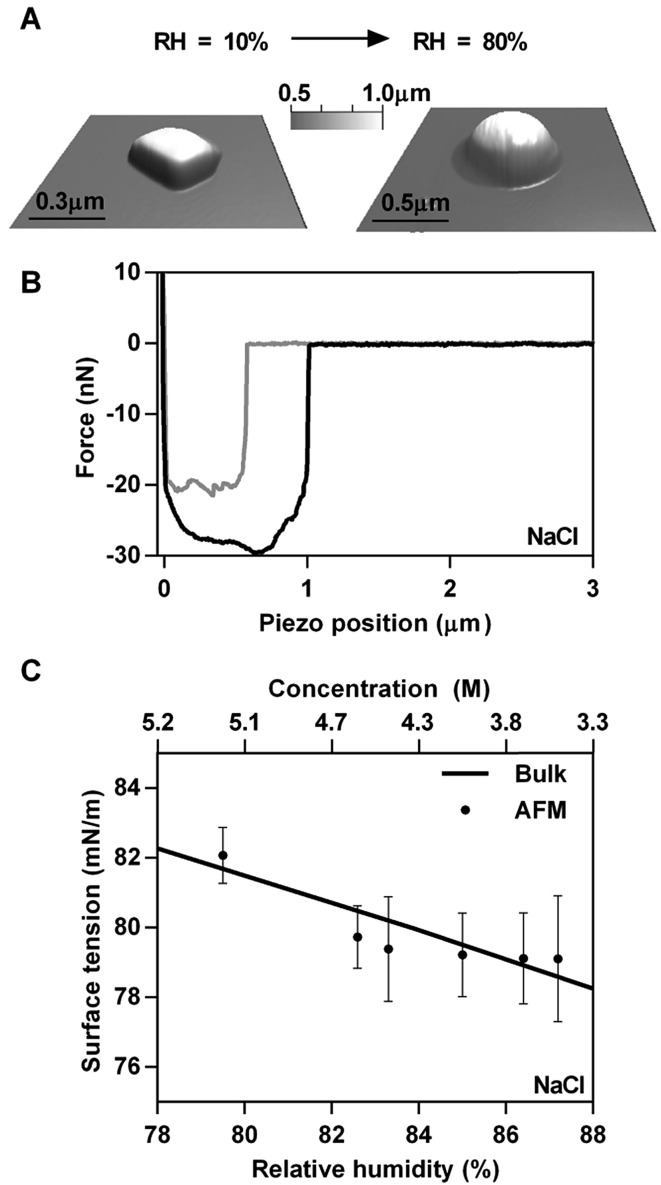
Experimental results of AFM based surface tension measurements of a ∼500 nm NaCl droplet. (A) 3D image of a solid NaCl crystal at 10% RH and deliquesced NaCl particle at 80% RH. (B) Experimental force plot on a NaCl droplet. The approach data is in grey and the retract data is in black. (C) Surface tension measurements (average and standard deviation) as a function of RH (bottom axis) and solute concentration (top axis). Predicted data is obtained from bulk solution surface tension measurements and is shown as the solid line.

The first system studied was NaCl. Fig. 2A shows 3D AFM images that demonstrate the phase transition of a solid NaCl particle at low RH (∼10%) to a liquid droplet at 80% RH. The observed phase transition is also apparent in the morphological change from a cubic NaCl crystalline solid to a round liquid droplet, which is approximately two times larger than the solid particle. The force plot in Fig. 2B is collected at the approximate centre of a NaCl droplet. While NaCl deliquesces at ∼75% RH,^13^ the surface tension measurements were complicated by the fact that a solid core was still observed in the force profile until ∼79% RH, which led to inconsistencies in the data. The presence of a solid core at RH above 75% has also been observed by others.^44^ At relatively high RH (above 88% in this case) the droplets become unstable under the mechanical force of the AFM cantilever during imaging. Consequently, imaging was performed initially under low RH to locate the particle and then minimally at higher RH to avoid damaging the droplet. Taking these issues into consideration, the range of RH probed for NaCl was between 78 and 88%.

At RH values above the deliquescence humidity of NaCl the deliquesced droplet continually takes up water, which effectively dilutes the salt concentration. Since surface tension is highly dependent on solute concentration,^10,11^ measurements of the retention force and determination of the surface tension using eqn (2) were performed as a function of RH. Fig. 2C shows experimentally determined surface tension values collected on several representative submicrometre size droplets as a function of RH (bottom axis) and concentration (top axis). For all model systems, 2–3 droplets were probed and at least 20 force measurements were performed at each RH. A noticeable change in the surface tension is observed (82–79 mN m^–1^), even for a relatively small change in RH, due to dilution of the NaCl upon the uptake of water, hence decrease in the concentration of solute with increasing RH. In order to verify that the single droplet AFM method is accurate, we compared our results to bulk surface tension measurements at concentrations relevant to the RH range that was probed. Water activity data were utilized to determine solute concentration at a particular RH^10^ (see ESI† for details) and the predicted relationship is represented in Fig. 2C as the solid line. The close overlap between the AFM-based surface tension measurements on individual submicrometre droplets with the bulk values provides strong evidence that AFM based tensiometer measurements are accurate and reliable. Predicted data obtained using the Extended AIM Aerosol Thermodynamic Model (AIM model)^45–49^ also display close overlap between bulk surface tension predictions and AFM measurements (see ESI†). The NaCl concentration range associated with the AFM surface tension measurements for the data shown in Fig. 2C is approximately 3–5 M, indicating that the droplets are highly concentrated and nearing their saturation solubility point.

### GA and MA AFM results

**Fig. 3:**
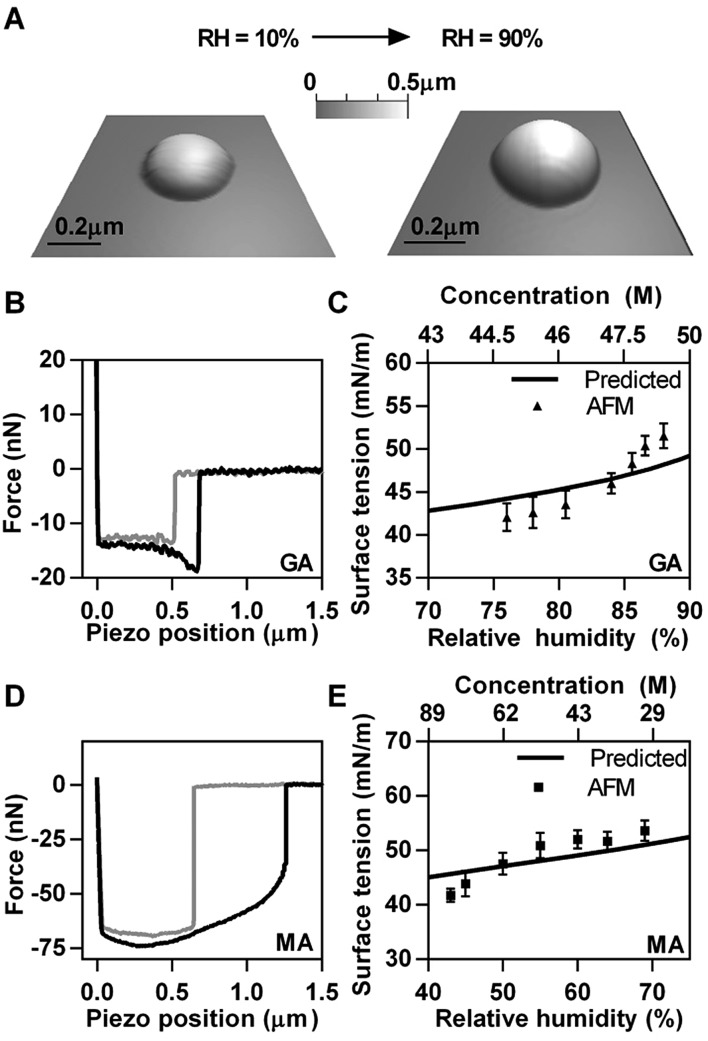
Experimental results of AFM based surface tension measurements of GA and MA. (A) 3D images of a solid GA particle at 10% RH and deliquesced GA particle at 90% RH. (B and D) Experimental force plots on GA (B) and MA (D) droplets. The approach data is in grey and the retract data is in black. (C and E) AFM based surface tension measurements (average and standard deviation) as a function of RH (bottom axis) and solute concentration (top axis) of GA (C) and MA (F). Predicted data (solid lines) are obtained from bulk solution surface tension measurements.

Having validated AFM-based surface tension measurements on NaCl salt, we extended our measurements to model organic systems, GA and MA, using a similar approach. Fig. 3A shows a typical GA particle at 10% RH and corresponding deliquesced droplet at 90% RH. Hence, as a result of water uptake, round amorphous GA particle with diameter of 0.7 μm and height of 0.25 μm (10% RH) became a liquid droplet with diameter of 0.9 μm and height of 0.4 μm (90% RH). The morphology of MA particles and submicrometre droplets are similar to that of GA (images are not shown). Experimental force plots collected on GA and MA droplets are shown in Fig. 3B and D, respectively. Both GA and MA droplets were similar in height (∼400 nm) at approximately 85% and 70% RH, respectively.

The RH range differed for each chemical system due to different hygroscopic properties. GA was probed in the range of approximately 75–90% RH, while MA was measured over the range of approximately 40–70% RH. Since GA does not absorb a significant amount of water until above 83%,^14^ the RH was first raised to 90% in order to deliquesce the particles and force measurements were taken during the dehydration cycle so that a larger range of RH could be probed.^50^ MA was measured on the hydration cycle since it steadily takes up water from relatively low RH.^14,50^ AFM surface tension measurements at different RH values for GA and MA are reported in Fig. 3C and E, respectively. The predicted surface tension dependence as a function of RH and concentration (solid lines in Fig. 3C and E) was obtained using bulk solution surface tension measurements as a function of solute concentration. However, since concentrations of the droplets measured with AFM were much higher than what is accessible by bulk measurements (due to solubility limits) the surface tension was extrapolated using a predictive model^51,52^ for highly concentrated solutions (details in ESI†).^53^ The water activity of GA and MA were obtained using the online modeling programs Aerosol Inorganic Mixtures Functional groups Activity Coefficient (AIOMFAC model)^14,54–56^ and the AIM model to establish the concentration of the droplets as a function of RH.^45–49^ The results of the AFM based surface tension for both GA and MA submicrometre droplets agree reasonably well both in terms of absolute values and with the predicted trend of surface tension as a function of RH due to changes in concentration upon uptake or release of water. We note, however, that both GA and MA show noticeable deviation from the bulk solution prediction at lower RH, which corresponds to higher solute concentrations. The origin of the deviation is likely due to non-ideal behavior of solutions at such high concentrations, which likely results in incorrect predictions obtained from the bulk solution data.

## Conclusions

This study provides a quantitative way to measure the surface tension of submicron size atmospherically relevant droplets under ambient pressure. Previously, there had been no reported methods that could directly probe the surface tension of submicron size droplets and furthermore, as a function of changing RH. The implications of this method, as well as subsequent future surface tension studies, are that the understanding of the role of atmospheric aerosols in cloud formation could be significantly improved and thus should advance the field of atmospheric chemistry and atmospheric science, as well as improve predictive power through more accurate models and theories that utilize surface tension. Now that the method has been shown to work on both organic and inorganic model systems, measurements can be performed on more complex multi-component systems, as well as authentic SSA and any water-soluble substrate deposited particles in the size range from ∼300 nm up to few micrometers. Nanoscale phenomena such as surface partitioning and size-effects can be addressed by directly probing surface tension and not just modeling it; as models typically assume ideal behavior which is often not the case for these highly concentrated solutions that are typical of atmospheric aerosol.
