# Correction: Humidity-dependent surface tension measurements of individual inorganic and organic submicrometre liquid particles

**DOI:** 10.1039/c5sc90044a

**Published:** 2015-08-10

**Authors:** Holly S. Morris, Vicki H. Grassian, Alexei V. Tivanski

**Affiliations:** a Department of Chemistry , University of Iowa , Iowa City , Iowa 52242 , USA . Email: vicki-grassian@uiowa.edu

## Abstract

Correction for ‘Humidity-dependent surface tension measurements of individual inorganic and organic submicrometre liquid particles’ by Holly S. Morris *et al.*, *Chem. Sci.*, 2015, **6**, 3242–3247.



## 

An error in Fig. 3 has been corrected; in particular, the top *x*-axis of plot “C” has been corrected by reversing the concentration scale so that values decrease from left to right. The corrected figure is shown below.

**Fig. 3:**
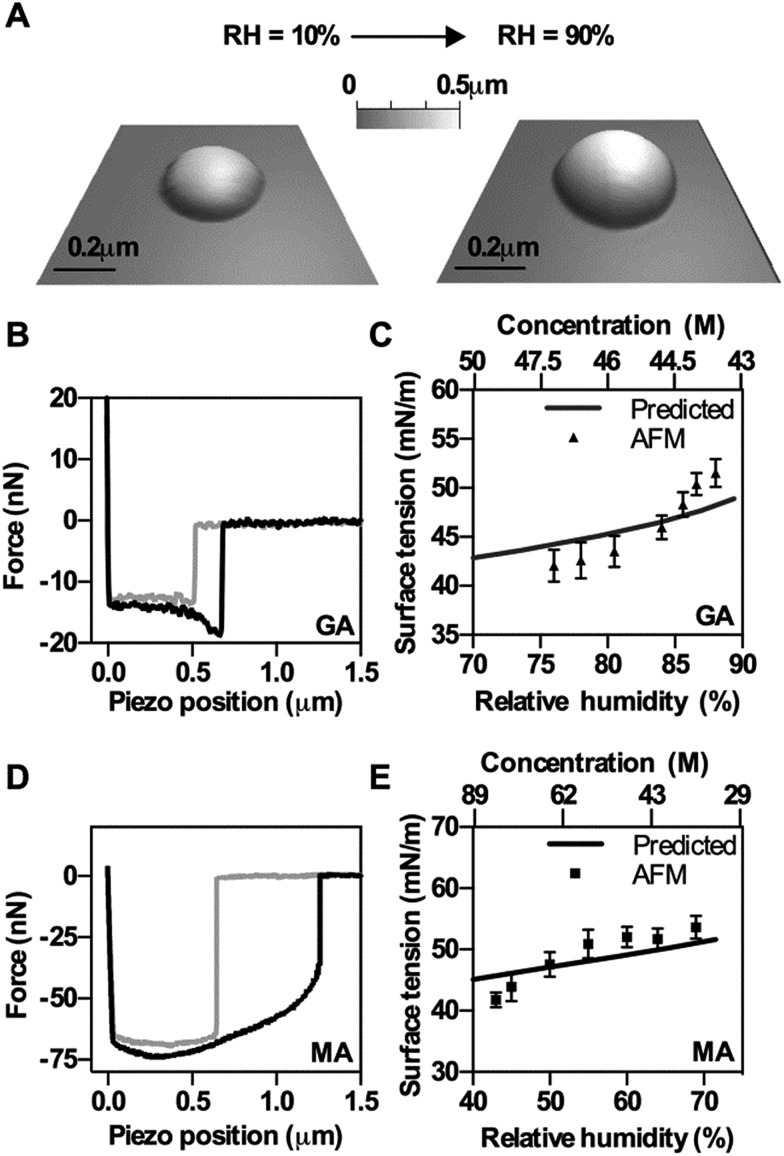
Experimental results of AFM based surface tension measurements of GA and MA. (A) 3D images of a solid GA particle at 10% RH and deliquesced GA particle at 90% RH. (B and D) Experimental force plots on GA (B) and MA (D) droplets. The approach data is in grey and the retract data is in black. (C and E) AFM based surface tension measurements (average and standard deviation) as a function of RH (bottom axis) and solute concentration (top axis) of GA (C) and MA (F). Predicted data (solid lines) are obtained from bulk solution surface tension measurements.

The Royal Society of Chemistry apologises for these errors and any consequent inconvenience to authors and readers.

