# Supplementary material for: Humidity-dependent surface tension measurements of individual inorganic and organic submicrometre liquid particles
Source: Chem Sci. 2015 Mar 31;6(5):3242–7. doi: 10.1039/c4sc03716b (PMC5490419; doi:10.1039/c4sc03716b)
Supplement: Supplementary file 1 [file SC-006-C4SC03716B-s001.pdf]

Electronic Supplementary Material (ESI) for Chemical Science.
This journal is © The Royal Society of Chemistry 2015

Electronic Supplementary Information

Humidity-Dependent Surface Tension Measurements of Individual Inorganic and Organic Submicrometre Liquid Particles

Holly S. Morris, Vicki H. Grassian* and Alexei V. Tivanski*

Department of Chemistry, University of Iowa, Iowa City, Iowa 52242.Email: vicki-grassian@uiowa.edu, alexei-tivanski@uiowa.edu

1. SEM images of nanoneedles

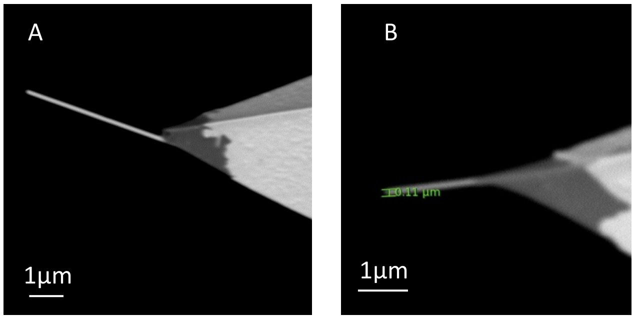

Figure 1S. A. SEM image (provided by Nauga Needles) of a constant diameter AgGa nanoneedle on the end of the AFM probe. B. Higher resolution SEM image showing the radius of the needle used to estimate r, which is used to calculate surface tension.

2. AFM bulk surface tension measurements

The nanoneedle spring constant was first calibrated on a clean mica surface. The petri dish with DI water was placed on the AFM stage and the tip was lowered to the water surface in AC mode with high amplitude, as to not submerge it in the liquid. The free amplitude was set to 3.0 V and target amplitude was 2.0 V. Once the target amplitude was reached, force plots were taken with no set point at the maximum starting distance and a large force distance of 5-6μm. The force plot showed no contact with liquid, so the starting distance was decreased until the force plot showed the profile indicative of touching the liquid surface. The retention force was used to calculate the surface tension of the water using equation 2. The water was then

transferred into a sample cup and the surface tension was measured once again with the conventional tensiometer.

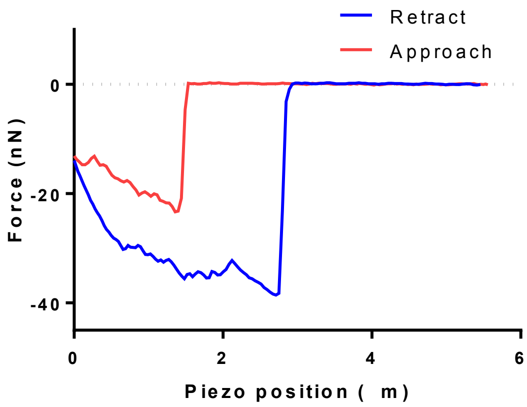

Figure 2S. Representative force plot of bulk surface tension experiment using AFM on DI water.

3. Predicting surface tension

As a reference for the AFM based surface tension measurements, theoretical predictions were made to estimate the surface tension of the highly concentrated droplets in the RH range measured with AFM. First, bulk tensiometer measurements were taken for several of concentrations of each chemical model system (Figures 3SA, 4SA, 5SA). NaCl surface tension as a function of concentration is linear and experimental data was fit with equation S1 (solid black line 3SA) where σ is surface tension, C is molar concentration of solute and σ_w is surface tension of water.[1] For GA and MA, the bulk surface tension measurements were fit with equation S2, where R is gas constant, T is temperature, Γ^{∞} is maximum surface excess, and b is inverse Langmuir adsorption constant.[1] Γ^{∞} was found in Spartan; 68.9 Å and 68.7Å for GA and MA, respectively. Value for b for GA and MA was found in the literature ($2.4*10^{-6}$) and ($2.5*10^{-6}$), respectively.[1] This data is represented as the solid black lines in 4SA and 5SA. Water activity for each species was then calculated (Figures 3SB, 4SB, 5SB). NaCl water activity was found by Tang et al., who reported the fit coefficients.[2] For GA and MA, the online modeling program, Aerosol Inorganic-Organic Mixture Functional groups Activity Coefficients (AIOMFAC) was used.[3-5] These data were then combined to obtain the relationship between surface tension and RH (Figures 3SC, 4SC, 5SC).

$$\sigma = \sigma_w + \frac{\Delta\sigma}{\Delta C}C \quad \text{(Eq. S1)} \qquad \sigma = \sigma_w - RT\Gamma^{\infty} ln\left(1 + \frac{C}{b}\right) \quad \text{(Eq. S2)}$$

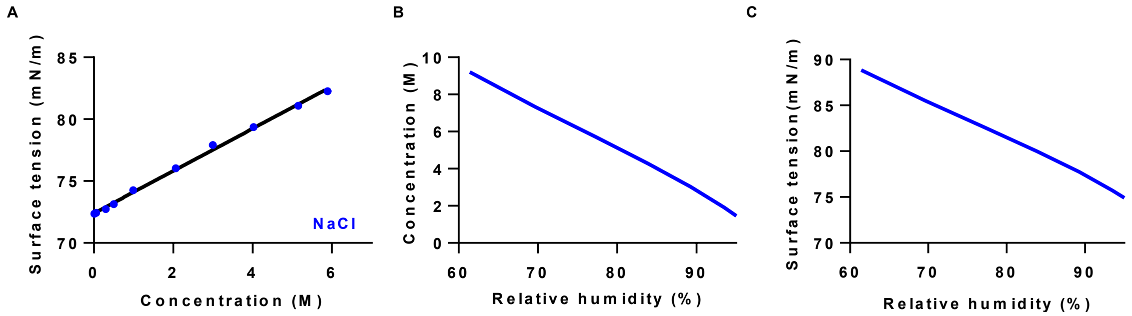

Figure 3S. Predicting surface tension dependence as a function of RH of NaCl. A. Bulk surface tension measurements as a function of solute molar concentration with linear fit (solid line). B. Solute concentration as a function of RH, as observed and reported by Tang et al.[2] C. Corresponding surface tension as a function of RH.

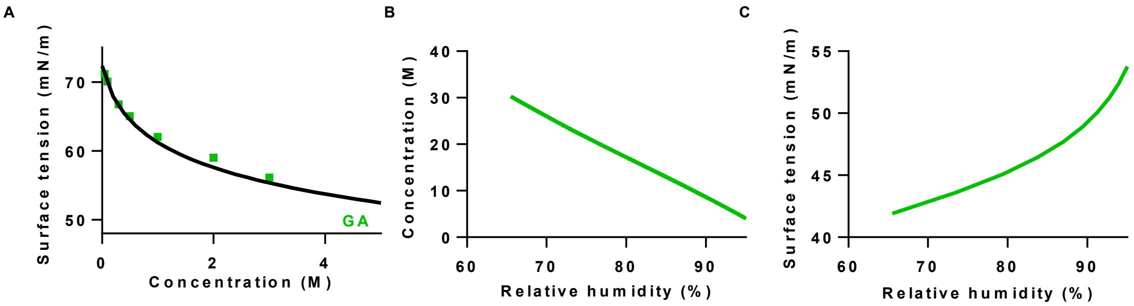

Figure 4S. Predicting surface tension a function of RH of GA. A. Bulk surface tension measurements as a function of solute molar concentration with fit to a semi-empirical model (solid line). B. The mass of water in the particle as a function of RH was found using the AIOMFAC model. This was converted to concentration and plotted versus RH. C. Corresponding surface tension as a function of RH.

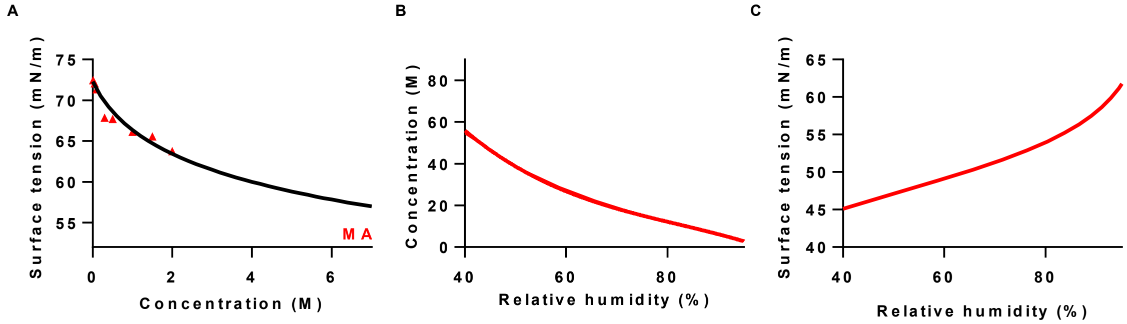

Figure 5S. Predicting surface tension as a function of RH of MA. A. Bulk surface tension measurements as a function of solute molar concentration with fit to a semi-empirical model (solid line). B. The mass of water in the particle as a function of RH was found using the AIOMFAC model. This was converted to concentration and plotted versus RH. C. Corresponding surface tension as a function of RH.

AIM prediction of NaCl surface tension

Figure 6S shows the results of AFM and bulk measurements compared to AIM predictions[6-8]; black data is our experimental data (AFM measurements and bulk solution surface tension measurements) and the green line is the relationship predicted with the AIM model. The AIM prediction is lower than our experimental data but still agrees well. The differences are probably due to the fact that surface tension/concentration (dσ/dM) values for NaCl, while studied very frequently, still has a large rang of reported values (1.46 – 2.08 mM dm^3 m^{-1} mol^{-1}) and can lead to discrepancies between predicted and experimental data, especially for highly concentrated solutions.[8] This could be due to chemical impurities. Since the same NaCl (Sigma-Aldrich, >99% purity, batch #007K0149) was used for both the bulk measurements and the AFM measurements, it is most likely the reason why our experimental data agrees better than with the AIM prediction.

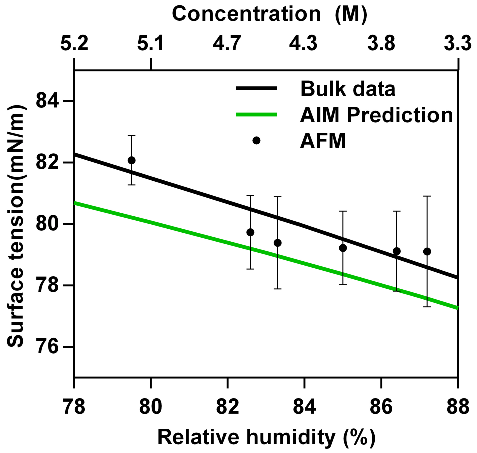

Figure 6S. Experimental AFM data and bulk solution measurements are in black. AIM predicted data is in green.

References

(1) Aumann, E.; Hildemann, L. M.; Tabazadeh, A. *Atmos Environ* **2010**, *44*, 329.
(2) Tang, I. N.; Tridico, A. C.; Fung, K. H. *J Geophys Res-Atmos* **1997**, *102*, 23269.
(3) Zuend, A.; Marcolli, C.; Booth, A. M.; Lienhard, D. M.; Soonsin, V.; Krieger, U. K.; Topping, D. O.; McFiggans, G.; Peter, T.; Seinfeld, J. H. *Atmos Chem Phys* **2011**, *11*, 9155.
(4) Zuend, A.; Marcolli, C.; Luo, B. P.; Peter, T. *Atmos Chem Phys* **2008**, *8*, 4559.
(5) 2014.
(6) Clegg, S. L.; Brimblecombe, P.; Wexler, A. S. *J Phys Chem A* **1998**, *102*, 2155.
(7) Clegg, S. L.; Pitzer, K. S.; Brimblecombe, P. *J Phys Chem-Us* **1992**, *96*, 9470.
(8) Dutcher, C. S.; Wexler, A. S.; Clegg, S. L. *J Phys Chem A* **2010**, *114*, 12216.
